# Predictors of adverse diastolic remodeling in non-diabetic patients presenting with ST-elevation myocardial infarction

**DOI:** 10.1186/s12872-023-03064-7

**Published:** 2023-01-23

**Authors:** Lawien Al Ali, Hilde E. Groot, Solmaz Assa, Erik Lipsic, Yoran M. Hummel, Dirk J. van Veldhuisen, Adriaan A. Voors, Iwan C. C. van der Horst, Carolyn S. Lam, Pim van der Harst

**Affiliations:** 1 Department of Cardiology, University Medical Center Groningen, University of Groningen, Hanzeplein 1, PO Box 30.001, 9700 RB Groningen, the Netherlands; 2 National Heart Centre Singapore, Duke-National University of Singapore, Singapore, Singapore; 3 Department of Critical Care, Maastricht University Medical Center, Maastricht, the Netherlands; 4 Department of Heart and Lungs, University Medical Center Utrecht, University of Utrecht, Utrecht, the Netherlands

**Keywords:** Diastolic function, Remodeling, ST-elevation myocardial infarction, Ischemic heart disease, Biomarkers

## Abstract

**Background:**

Adverse systolic remodeling after ST-elevation myocardial infarction (STEMI) is associated with poor clinical outcomes. However, little is known about diastolic remodeling. The purpose of this study was to identify the factors leading to diastolic remodeling.

**Methods:**

Echocardiography was performed during hospitalization and at 4 months follow-up in 267 non-diabetic STEMI patients from the GIPS-III trial. As parameters of diastolic remodeling we used (1.) the E/e′ at 4 months adjusted for the E/e′ at hospitalization and (2.) the change in E/e′ between hospitalization and 4 months. Multivariable regression models correcting for age and sex were constructed to identify possible association of clinical and angiographic variables as well as biomarkers with diastolic remodeling.

**Results:**

Older age, female gender, hypertension, multi vessel disease, higher glucose and higher peak CK were independent predictors of higher E/e′ at 4 months in a multivariable model (R^2^:0.20). After adjustment for E/e′ during hospitalization only female gender, multivessel disease and higher glucose remained predictors of E/e′ at four months (R^2^:0.40). Lower myocardial blush grade, AST and NT-proBNP were independent predictors of a higher increase of E/e′ between hospitalization and at 4 months in a multivariable model (R^2^:0.08).

**Conclusions:**

Our data supports the hypothesis that female gender, multivessel coronary artery disease, and microvascular damage are important predictors of adverse diastolic remodeling after STEMI. In addition, our data suggests that older age and hypertension prior to STEMI may have contributed to worse pre-existing diastolic function.

*Trial registration*: NIH, NCT01217307. Prospectively registered on October 8th 2010, https://clinicaltrials.gov/ct2/show/NCT01217307.

**Supplementary Information:**

The online version contains supplementary material available at 10.1186/s12872-023-03064-7.

## Background

Myocardial damage caused by myocardial infarction (MI) can lead to adverse cardiac remodeling which is associated with the development of heart failure and malignant arrhythmias [1]. Heart failure after MI is often characterized by reduced left ventricular systolic and diastolic dysfunction [2, 3]. Initial studies on cardiac remodeling after MI were primarily focused on left ventricular systolic function, its predictors and prognostic implications for outcome [4, 5]. Our understanding and knowledge of diastolic function after MI is still lacking [4]. Several studies have suggested that diastolic dysfunction, in the absence of systolic dysfunction, after MI is also associated with morbidity and mortality [2, 6]. Furthermore, approximately half of patients presenting with clinical heart failure after MI have diastolic dysfunction with preserved systolic function [2, 4, 7]. Adverse diastolic remodeling, defined as progression of diastolic dysfunction after MI, is emerging as an important prognostic marker for outcome [8].

Several risk factors, notably hypertension and diabetes, have been identified as predictors of diastolic dysfunction in the general population [9, 10]. However, little is known about the elements that are specifically related with diastolic function following myocardial remodeling after MI. Therefore, the aim of this study was to identify the factors associated with adverse diastolic remodeling after MI, specifically in the setting of ST-elevation myocardial infarction.

## Methods

The current study is a predefined echocardiography sub-study of the GIPS-III trial. This randomized, double blind, placebo-controlled trial studied the effect of metformin on left ventricular ejection fraction (LVEF) in patients presenting with STEMI. Design of this study, baseline patient characteristics, primary outcomes and effect on diastolic function have been reported previously. In summary, between January 1, 2011 and May 26, 2013, patients presenting to the University Medical Center Groningen (UMCG) with STEMI who underwent successful PCI with implantation of at least 1 stent with a diameter of at least 3 mm were included. Important exclusion criteria were age younger than 18 years, previous myocardial infarction, known diabetes, inability to undergo magnetic resonance imaging and severe renal dysfunction. Patients were randomized to either metformin 500 mg twice daily or visually matching placebo twice daily, which started immediately after PCI. After 4 months of treatment patients underwent cardiac MRI to assess the primary endpoint, LVEF. Treatment duration of 4 months was chosen based on the presumption that the majority of myocardial remodeling occurs over the course of this critical period [11, 12]. Our research showed that metformin treatment, when compared to placebo, did not improve LV systolic or diastolic function at four months follow-up [11, 13, 14].

For the current study, all patients from the GIPS-III study with reliable measurements of the ratio of transmitral early flow to early mitral annulus velocity (E/e′) were included.

### Baseline parameters

Venous blood samples were taken at admission for standard blood analyses at the central laboratory of the UMCG. Blood analyses were repeated during hospitalization to determine enzymatic infarct size. The variables collected were age, gender, history of hypertension, history of hypercholesterolemia, smoking status, total ischemic time, heart rate, systolic blood pressure, diastolic blood pressure, hemoglobin, thrombocytes, leucocytes, aspartate aminotransferase (AST), alanine aminotransferase (ALT), total cholesterol, high-density lipoproteins, low-density lipoproteins, triglycerides, sodium, potassium, creatinin, ureum, glucose, glycated hemoglobin (HbA_1c)_, total creatine kinase (CK-total), myocardial band creatine kinase (CK-MB), N-terminal prohormone of brain natriuretic peptide (NT-proBNP), high sensitive troponin T. Periprocedural angiographic assessments included: absence or presence of multi vessel disease, thrombolysis in myocardial infarction (TIMI) grade flow before reperfusion therapy, TIMI grade flow after reperfusion therapy and myocardial blush grade (MBG) after reperfusion therapy.

### Echocardiography

Trans-thoracic echocardiogram was performed during hospitalization for the index event and at four months follow-up. The protocol for obtainment of the echocardiographic exams has been reported previously [14]. Based on current guidelines, the following structural measurements were assessed: Left ventricular (LV) interventricular septum and posterior wall thickness (IVS and LVPW), left ventricular end-diastolic (LVEDD) and end-systolic diameter (LVESD) [15, 16]. In addition, Simpson’s biplane volumetric parameters including LV end-diastolic volume (LVEDV) and end-systolic volume (LVESV) were measured. LVEF was calculated as LVEF = (LVEDV-LVESV)/LVEDV × 100%. Left atrial volume (LAV) was measured with the area length method. The LV mass was estimated from linear dimensions as suggested by Devereux and colleagues [17]. Parameters for LV diastolic function included Doppler measurement of the mitral valve early filling flow (E), active atrial filling flow (A), isovolumetric relaxation time (IVRT) and E wave deceleration time (DT) as well as tissue color Doppler measurements of early diastolic tissue velocities (e′) from both the septal and lateral wall were measured [18, 19]. Mean e′ was calculated as (e′ septal + e′ lateral)/2. E/eˈ was calculated as E/mean e′. Reported values represent the mean of three heart beats in end-expiration.

### Diastolic function and diastolic remodeling

Diastolic remodeling was considered to be the change in diastolic function during the critical period after STEMI in which myocardial remodeling is thought to occur. For the evaluation of the diastolic function E/e′ was applied, as it has a strong correlation with invasively measured cardiac filling pressure [20, 21]. Our primary parameters for post-STEMI diastolic remodeling were (1.) the E/e′ at 4 months adjusted for the E/e′ at hospitalization and (2.) the change in E/e′ between hospitalization and 4 months.

### Statistical analysis

Continuous variables are presented as mean ± standard deviation (SD) or median (interquartile range, IQR) for normally and non-normally distributed data, respectively. Non-normally distributed data was transformed using log-transformation. Outcome observations of median ± 5 times the standard deviation were considered extreme outliers and excluded from the cohort. Differences between groups were tested using two-tailed t test for normally distributed data and Wilcoxon rank-sum for non-normally distributed data. To identify possible association of clinical and angiographic variables as well as biomarkers with diastolic function at follow-up, multivariable regression models correcting for age and sex were constructed using our baseline parameters as well as LVEF during hospitalization. Linear regression was applied for outcome variables on a continuous scale and logistic regression was applied for binary outcome variables. The candidate sets of variables measured at baseline (*P* < 0.10) were checked for correlation and when appropriate the variable with the weakest relation to the outcome was excluded from further analysis. Bootstrapping stepwise regression was used to narrow the remaining candidate set of variables that were associated with E/e′ at four-month follow-up. The bootstrap sample size was 266 (99.6% of the entire data set). Variables selected > 600 times were assumed to be accurate and included in the final multivariable model. The final multivariable predictive model for E/e′ at follow-up was adjusted for the E/e′ at hospitalization to identify variables that were associated with diastolic remodeling. All reported *P* values are two-sided, and a *P* value of < 0.05 was considered to indicate an independent association. All analyses were performed using Stata version 14.0 (StataCorp).

## Results

### Study population

**Fig. 1:**
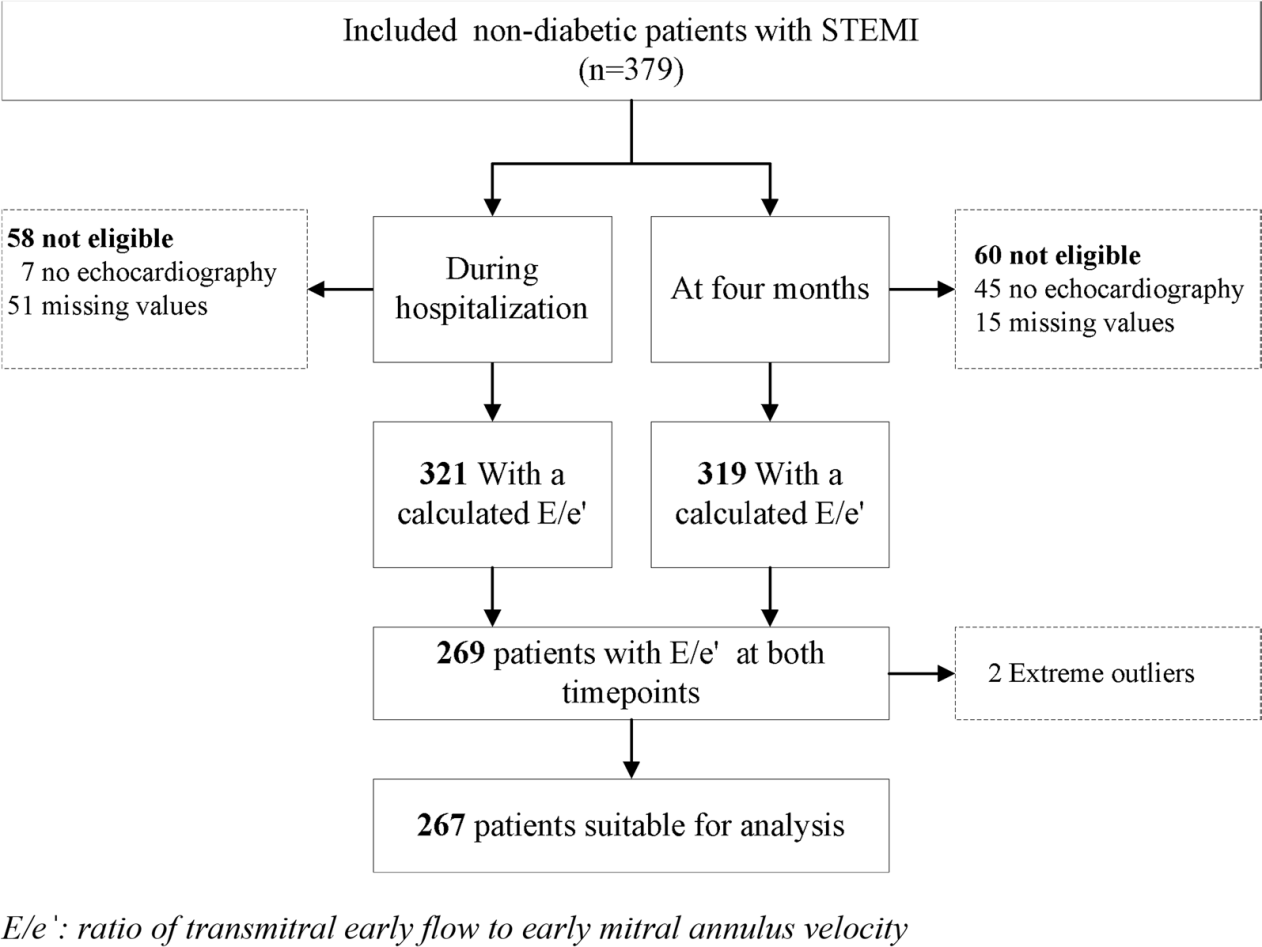
Flowchart of patient population. E/eˈ: ratio of transmitral early flow to early mitral annulus velocity

All 379 patients participating in the GIPS-III trial were alive at four months. 329 (87%) patients underwent echocardiography both during hospitalization and at four months; 43 (11%) of these patients only underwent echocardiography during hospitalization and 5 (1.3%) patients only at four months. E/e′ could be determined in 321 (85%) patients during hospitalization and in 319 (84%) patients at four months. 269 (71%) patients with measurements of E/e′ at both time points were eligible for the study analysis. Of these, 2 (1%) patients were recognized as extreme outliers and excluded from the cohort 267 (70%) patients with reliable measurement of E/e′ remained eligible for the final analysis (Fig. 1). Median E/e′ were 7.51 (IQR 6.14, 9.60) at hospitalization and 7.53 (IQR 6.37, 8.77) at 4 months. Average time between the myocardial infarction and echocardiographic evaluation was two days during the initial hospitalization and 124 days at follow-up.

Mean age of the 267 included patients was 58.0 (± 11.3) years, 23% were females and 38% had an infarction of the left anterior descending artery. The baseline and hospitalization characteristics were compared between the patients with E/e′ at 4 months follow up above or below median (Table 1). There were more women in the group with an E/e′ at 4 months above median (31.6%) as compared to the group with an E/e′ at 4 months below median (14.9%; *P* < 0.01). Patients with an E/e′ at 4 months above median were older, had a higher systolic blood pressure and were more likely to have a history of hypertension. Plasma level of glucose and NT-proBNP at baseline were also higher in patients with an E/e′ at 4 months above median. There were no differences in the prescription of cardiac medication at discharge between the two groups (Additional file 1: Table S1).

**Table 1:** Baseline characteristics of all patients, and stratified by E/e′ below and above median at 4 months after STEMI

		No. (%)		
Characteristic	Total (n = 267)	E/e′ ≤ median (n = 134)	E/e′ > median (n = 133)	*P *value
Age, years	58.0 ± 11.3	56.2 ± 10.6	59.9 ± 11.7	< 0.01
Women	62 (23.2%)	20 (14.9%)	42 (31.6%)	< 0.01
Metformin treatment	139 (52.1%)	68 (50.7%)	71 (53.4%)	0.67
Body weight, kg	84.3 ± 14.5	85.6 ± 13.6	82.9 ± 15.3	0.12
Body-mass Index, kg/m^2^	26.8 ± 3.6	26.8 ± 3.5	26.8 ± 3.6	0.85
*Race/ethnicity*				
White	257 (96.3%)	131 (97.8%)	126 (94.7%)	0.19
Asian	9 (3.4%)	3 (2.2%)	6 (4.5%)	0.30
Black	1 (0.4%)	0 (0.0%)	1 (0.8%)	0.50
*Cardiovascular related history*				
Hypertension	73 (27.3%)	28 (20.9%)	45 (33.8%)	0.02
Dyslipidemia	163 (61.0%)	83 (61.9%)	80 (60.2%)	0.76
Current smoking	145 (54.3%)	79 (59.0%)	66 (49.6%)	0.13
Stroke	2 (0.7%)	1 (0.7%)	1 (0.8%)	1.00
Previous PCI	3 (1.1%)	2 (1.5%)	1 (0.8%)	0.57
*Blood pressure, mmHg*				
Systolic	134.6 ± 23.2	131.2 ± 20.9	138.0 ± 24.8	0.02
Diastolic	84.8 ± 14.5	83.6 ± 14.0	86.0 ± 15.0	0.19
Heart rate, beats/min	75.6 ± 15.6	76.0 ± 16.2	75.3 ± 15.0	0.71
*Infarct-related factors*				
Ischemia time, min	154 (108, 250)	150 (109, 240)	161 (108, 254)	0.50
Multi vessel disease	80 (30.0%)	34 (25.4%)	46 (34.6%)	0.10
Anterior infarction	102 (38.2%)	48 (35.8%)	54 (40.6%)	0.42
*Intervention-related assessments*				
TIMI flow grade pre PCI ≤ 1	173 (64.8%)	87 (64.9%)	86 (64.7%)	0.96
TIMI flow grade post PCI < 3	20 (7.5%)	10 (7.5%)	10 (7.5%)	0.99
Myocardial blush grade ≤ 1	24 (9.1%)	11 (8.3%)	13 (9.8%)	0.67
*Laboratory values at admission*				
Glucose, mmol/ L	8.1 (7, 9.5)	7.9 (6.9, 8.9)	8.5 (7, 10.1)	0.03
HbA_1c_, %	5.8 (5.6, 6)	5.7 (5.6, 5.9)	5.8 (5.6, 6.1)	0.07
Hemoglobin, mmol/ L	9 (8.4, 9.4)	8.95 (8.5, 9.4)	9 (8.4, 9.4)	0.93
Creatinine, µmol/ L	72 (63, 82)	74 (64, 83)	70 (61, 80)	0.07
eGFR, ml/min/1.73m^2^	95 (86, 103)	96 (86, 103)	94 (85, 103)	0.39
AST, U/L	28 (22, 41)	26 (22, 40)	31 (23, 45)	0.22
ALT, U/L	25 (18, 35)	24.5 (19, 33)	25 (17, 37)	0.61
NT-proBNP, ng/L	74 (37, 173)	59 (32, 110)	89 (45, 266)	< 0.01
CK, U/L	128 (84, 201)	132 (86, 201)	118 (81, 196)	0.73
Myocardial band of CK, U/L	16 (13, 25)	15 (13, 22)	16 (13, 30)	0.19
Troponine T, ng/L	46 (21, 135)	40 (20, 130)	52 (26, 136)	0.25
Total cholesterol, mmol/ L	5.4 (4.8, 6.1)	5.5 (4.8, 6.2)	5.3 (4.7, 6)	0.13
LDL cholesterol, mmol/ L	3.8 (3.3, 4.4)	3.9 (3.3, 4.6)	3.8 (3.2, 4.4)	0.27
HDL cholesterol, mmol/L	1.1 (0.9, 1.3)	1.1 (0.9, 1.3)	1.1 (0.9, 1.4)	0.57

*E/e′* ratio of transmitral flow velocity (E) to early mitral annulus velocity (e′), *PCI* percutaneous coronary intervention, *TIMI* Thrombolysis in Myocardial Infarction, *HbA1c* glycated hemoglobin, *NT-proBNP* N-terminal pro brain natriuretic peptide, *eGFR* estimated glomerular filtration rate, *AST* Aspartate transferase, *ALT* Alanine transferase, *CK* creatine kinase, *LDL* low density lipoprotein, *HDL* high density lipoprotein

### Echocardiographic evaluation

In patients with an E/e′ at 4 months above median, echocardiography during hospitalization showed higher early filling flows, higher E/e′ ratio, lower diastolic tissue velocities, and smaller LVEDD (Table 2). In addition, at their 4-month evaluation patients with an E/e′ above median had higher LVESV, E, A, E/A ratio, and LAVI and lower LVEF, and diastolic tissue velocities in their echocardiograph as compared to patients with an E/e′ below median (Table 2).

**Table 2:** Echocardiographic measurements during hospitalization, at 4 months and the changes between both time points stratified by E/e′ at 4 months after STEMI

	During hospitalization			At 4 months			Change between both visits		
Variable	E/e’ ≤ median (n = 134)	E/e′ > median (n = 133)	*P *value	E/e′ ≤ median (n = 134)	E/e′ > median (n = 133)	*P *value	E/e′ ≤ median (n = 134)	E/e′ > median (n = 133)	*P *value
Time until echo, days	2 (1, 3)	2 (1, 3)	0.26	124 (119, 129)	123 (116, 128)	0.41	121 (114, 126)	121 (113, 126)	0.98
LVEDV, ml	103.2 ± 26.9	100.6 ± 30.0	0.49	105.4 ± 28.2	111.4 ± 34.9	0.14	3.9 ± 21.5	12.2 ± 24.3	< 0.01
LVESV, ml	50.4 ± 18.1	50.5 ± 21.5	0.98	46.3 ± 17.0	52.7 ± 24.2	0.02	− 2.0 ± 12.1	2.8 ± 15.2	0.01
LVEF, %	52 ± 9.4	51 ± 9.5	0.45	57 ± 7.4	54 ± 8.9	0.01	3.6 ± 7.7	3.3 ± 7.7	0.76
LV mass, gram	182.8 ± 49.9	175.5 ± 50.5	0.24	176.7 ± 45.5	179.9 ± 45.3	0.56	− 5.8 ± 38.7	3.3 ± 42.9	0.08
E, cm/s	62.9 ± 14.9	69.3 ± 19.5	< 0.01	62.1 ± 12.4	76.0 ± 17.4	< 0.01	− 0.8 ± 16.0	6.8 ± 18.9	< 0.01
A, cm/s	60.4 ± 14.2	69.8 ± 17.9	< 0.01	63.4 ± 13.5	70.8 ± 18.3	< 0.01	3.0 ± 11.6	1.0 ± 14.7	0.23
E/A ratio	1.1 ± 0.3	1.0 ± 0.4	0.28	1.0 ± 0.3	1.2 ± 0.5	< 0.01	− 0.1 ± 0.3	0.1 ± 0.4	< 0.01
DT, ms	184.8 ± 52.6	184.9 ± 56.5	1.00	219.4 ± 55.5	207.5 ± 67.3	0.12	34.6 ± 58.7	22.6 ± 78.6	0.16
IVRT, cm/s	98.9 ± 24.3	99.4 ± 26.6	0.88	100.5 ± 22.9	101.5 ± 21.5	0.76	− 0.2 ± 28.6	3.1 ± 24.9	0.49
Septal e′, cm/s	8.3 ± 2	7.1 ± 1.9	< 0.01	8.7 ± 1.9	7.4 ± 2	< 0.01	0.3 ± 2.1	0.2 ± 1.8	0.71
Lateral e′, cm/s	10 ± 3.1	8.6 ± 2.8	< 0.01	11 ± 2.5	8.9 ± 2.7	< 0.01	0.8 ± 2.9	0.3 ± 2.4	0.18
Mean e′, cm/s	9.4 ± 2.3	7.9 ± 2.1	< 0.01	10 ± 1.8	8.2 ± 2.2	< 0.01	0.5 ± 2.0	0.3 ± 1.7	0.26
E/e′ ratio	6.9 ± 1.9	9.2 ± 2.9	< 0.01	6.3 ± 0.9	9.6 ± 2.3	< 0.01	− 0.7 ± 1.8	0.5 ± 2.6	< 0.01
LAVI, ml/m^2^	27.3 ± 6.8	28.1 ± 7.6	0.39	28 ± 7.7	31 ± 8.7	0.02	1.3 ± 6.5	2.8 ± 7.3	0.11
LVMI, gram/m^2^	89.4 ± 22.2	89.8 ± 21.8	0.89	84.6 ± 21.8	89.4 ± 23.7	0.09	− 4.5 ± 21.8	− 1.3 ± 19.9	0.23
IVS, mm	11 ± 2	11 ± 1.9	0.10	10 ± 1.7	10 ± 1.8	0.55	− 0.3 ± 2.1	− 0.7 ± 1.9	0.18
LVPW, mm	9.7 ± 1.5	10 ± 3.2	0.28	9.3 ± 1.4	9.4 ± 1.5	0.95	− 0.4 ± 1.7	− 0.7 ± 3.4	0.26
LVEDD, mm	49 ± 5.1	47 ± 6.0	0.01	49 ± 5.5	50 ± 5.4	0.70	0.7 ± 4.8	2.6 ± 5.0	< 0.01
LVESD, mm	33 ± 5.6	32 ± 5.8	0.19	33 ± 5.8	34 ± 6.1	0.21	− 0.4 ± 6.1	1.3 ± 5.3	0.02

*LVEDV* left ventricular end-diastolic volume, *LVESV* left ventricular end-systolic volume, *LVEF* left ventricular ejection fraction, *LV mass* left ventricular mass, *E* passive early filling of the left ventricle, *A* active atrial filling of the left ventricle, *DT* E wave deceleration time, *IVRT* isovolumetric relaxation time, *Septal e′* early diastolic tissue velocity from septal wall, *Lateral e′* early diastolic tissue velocity from lateral wall, *LAVI* left atrial volume indexed for body mass, *LVMI* left ventricular mass indexed for body mass, *IVS* interventricular septal wall thickness, *LVPW* left ventricular posterior wall thickness, *LVEDD* left ventricular end-diastolic diameter, *LVESD* left ventricular end-systolic diameter

When comparing the changes between hospitalization and at 4 months, a larger increase in LVESV, LVEDV, LVESD, LVEDD, E, E/A ratio and E/e′ ratio was observed in the group with E/e′ above median (Table 3).

**Table 3:** Age and gender adjusted association of candidate baseline markers with E/e′ at 4 months after STEMI and change in E/e′ between hospitalization and follow-up

Predictor	E/e′ at follow-up			Change in E/e’		
	β	SE	*P *value	β	SE	*P *value
Hypertension	0.97	0.32	< 0.01			
Systolic blood pressure, mm/Hg per 10	0.13	0.06	0.03			
Multivessel disease	1.10	0.30	< 0.01			
Myocardial blush grade ≤ 1				1.25	0.49	0.01
Glucose, mmol/L	0.20	0.07	< 0.01			
HbA_1c_, %	0.47	0.27	0.08			
Potassium, nnol/L	− 0.74	0.39	0.05			
AST, U/L				− 0.97	0.29	< 0.01
LDH, U/L	0.37	0.19	0.06	− 0.56	0.19	< 0.01
CK-total, U/L per doubling	0.28	0.12	0.03	− 0.28	0.12	0.02
CK-MB, U/L per doubling				− 0.31	0.13	0.02
NT-proBNP, ng/L per doubling	0.23	0.09	0.01	− 0.18	0.09	0.04
Peak CK-total, U/L per doubling	0.24	0.08	< 0.01	− 0.16	0.08	0.07
Peak CK-MB, U/L per doubling	0.26	0.10	0.01	− 0.19	0.10	0.05
Peak NT-proBNP, ng/L per doubling	0.17	0.10	0.09			

*β* Beta, *SE* Standard error, *HbA*_*1c*_ Glycated hemoglobin, *AST* Aspartate transferase, *LDH* Lactate dehydrogenase, *CK* Total creatine kinase, *CK-MB* Myocardial band of creatine kinase, *NT-proBNP* N-terminal pro-brain natriuretic peptide

### Risk factors associated with diastolic remodeling

Multivariable linear regression analyses of the baseline variables showed an age and gender independent positive correlation between E/e′ at 4 months with hypertension, multivessel disease, systolic blood pressure, glucose, NT-proBNP, peak CK-total and peak CK-MB, a negative correlation with potassium, and a positive trend with HbA1c and peak NT-proBNP (Table 3). In a bootstrapped model for E/e′ at 4 months age, gender, hypertension, multivessel disease, glucose, peak CK remained highly selected. The multivariable adjusted association of these variables is presented in Table 4 (R^2^:0.20). When adjusting this model for E/e′ measured during hospitalization, only gender, multivessel disease and glucose remained associated with E/e′ at 4 months (Table 4; R^2^:0.40) suggesting that the effects of age, hypertension and infarct size might originate from prior to the measurements at hospitalization.

**Table 4:** Multivariate prediction models E/e′ at 4 months after STEMI and change in E/e′ between hospitalization and follow-up

Predictor	Multivariate model for E/e′ at follow-up			Multivariate model for E/e′ at follow-up corrected for E/e′ in hospital			Multivariate model for change in E/e’		
	β^a^	SE^b^	*P *value	β^a^	SE^b^	*P *value	β^a^	SE^b^	*P *value
Age, years	0.15	0.01	0.01	0.03	0.01	0.56			
Female gender	0.24	0.32	< 0.01	0.14	0.28	0.01			
Hypertension	0.14	0.31	0.01	0.07	0.27	0.16			
Multivessel disease	0.21	0.29	< 0.01	0.14	0.26	< 0.01			
Myocardial blush grade ≤ 1							0.18	0.48	< 0.01
Glucose, mmol/L	0.15	0.06	0.01	0.11	0.06	0.03			
AST^c^, U/L per 100							− 0.19	0.28	< 0.01
Peak CK-total^d^, U/L per doubling	0.15	0.08	0.01	0.03	0.07	0.56			
NT-proBNP^e^, ng/L per doubling							− 0.15	0.08	0.02

*β* Beta, *SE* Standard error, *AST* Aspartate transferase, *CK* Total creatine kinase, *NT-proBNP* N-terminal pro-brain natriuretic peptide

Analysis of the change in E/e′ between hospitalization and follow-up revealed an age and gender independent association of lower myocardial blush grade, AST, LDH, CK-total, CK-MB, NT-proBNP, and peak CK-MB with greater increase in E/e′, and a similar trend for peak CK-total (Table 3). The multivariable model for change in E/e′ included myocardial blush grade, AST and NT-proBNP (Table 4; R^2^:0.08).

Treatment allocation was not related to diastolic remodeling in either of the analyses.

## Discussion

In this predefined echocardiographic sub study of the GIPS-III trial we found that female gender, multivessel disease, higher glucose, lower blush-grade but also lower AST and lower NT-proBNP were independently associated with adverse diastolic remodeling after STEMI.

Our data adds to our understanding of cardiac remodeling after STEMI. Several previous studies reported on the factors associated with the development of reduced systolic function and identified infarct size and inflammatory markers as major determinants of systolic function after STEMI [22, 23]. Whether the same or other factors account for diastolic remodeling has not yet been established. In our study we found that deterioration of diastolic function after STEMI is more pronounced in women as compared to men. Interestingly, a previous meta-analysis of restrictive filling after acute MI found no difference in prevalence between men and women. However, in this study only diastolic function in the first days after MI and not diastolic remodeling was assessed. Also, a sex-specific study on the incidence and risk of new-onset heart [6] failure in 8592 subjects, found women to have a higher incidence of heart failure with preserved ejection fraction than men [24]. Our study is the first to report that female gender is an important predictor of adverse diastolic remodeling. Previous research has shown 17β-estradiol (E2) to have attenuating effects on oxidative stress and inflammatory processes [25], both known to be involved in the development of heart failure with preserved ejection fraction. The postmenopausal decline of E2 might explain why women are more at risk for adverse diastolic remodeling. Unfortunately, menopausal status and serum estrogen levels were not included in the GIPS-III data collection. However, a median age of 61 years for female patients in our cohort suggests that a vast majority of these women were postmenopausal at the time of their index event.

Ischemia related factors have previously been reported to increase left ventricular diastolic chamber stiffness with resulting increase of diastolic pressures [26]. Plasma levels of AST are a known marker of liver function. However, a significant portion of AST plasma levels is also derived from heart and other tissues. AST, like NT-proBNP, is associated with enzymatic infarct size and is a predictor of short- and long-term outcomes after STEMI [27–29]. In our study we found that conventional markers of enzymatic infarct size (CK, CK-MB) as well as AST and NT-proBNP were positively correlated with worse diastolic function at four months after STEMI. However, this association was no longer present when adjusting for the diastolic function measured during hospitalization, suggesting that the adverse effects of infarct size on diastolic function is already present during hospitalization. In our additional analyses of diastolic remodeling (change in E/eˈ) we observed that larger enzymatic infarct size did not lead to further deteriorate of diastolic function between hospitalization and at four months, but rather, tended to improve. A previous study on diastolic remodeling as a prognostic indicator found adverse diastolic remodeling to be associated with worse outcome and also reported a correlation between adverse diastolic remodeling and myocardial infarction size [8]. In contrast to our results, their study found a positive correlation between adverse diastolic remodeling and infarct scar size. Unlike our own study they did not investigate other parameters for possible correlation with diastolic remodeling. Direct comparison of results from different studies is hindered by differences in methods to measure diastolic function, applied definitions and interval between measurements. We defined adverse diastolic remodeling on a continuous scale, to increase power, as opposed to an arbitrary change in the diastolic dysfunction grade. Also, enzymatic infarct size and scar tissue are fundamentally different. Though both variables are markers for infarct size, scar tissue relates more to the amount of tissue lost while enzymatic infarct size markers relate closer to the amount of tissue damaged. Our findings are in line with previous studies which showed that diastolic function deteriorates during ongoing ischemia and that this deterioration can be partly reversible after reperfusion in case of diastolic stunning [26, 30–32]. Furthermore, our study was not limited to just the incidence occurrence but also aimed to identify factors that influence the deterioration of diastolic function after the acute phase of an infarction. In our study we found lower MBG to be associated with adverse diastolic remodeling. This finding supports the theory that microvascular abnormalities cause further diastolic deterioration after reperfusion. Prolonged coronary occlusion can result in loss of anatomic integrity in the microvascular network and abnormalities in coronary microvascular function are known to cause ischemia even in the absence of epicardial stenosis [33, 34]. Diabetes mellitus is also a well-established cardiovascular risk factor for the development of diastolic dysfunction [9, 10]. Although we excluded diabetic patients from our study, our findings still show that an increased plasma glucose level is associated with worse diastolic function. This is in accordance with previous findings that diastolic function is associated with glucose metabolism status even in prediabetics [35].

In our results we report that LVEDD during hospitalization was higher in patients with E/e′ below the median. However, there was no difference in LVEDV during hospitalization between patients with E/e′ above or below the median even though LVEDD and LVEDV both provide an indication of LV size. Similarly, we report LVESV at follow-up to be higher in patients with an E/e′ above the median, while no difference was found in LVESD. Though these results seem conflicting it must be stated that diameters are obtained from a single dimension while volumes are obtained from two dimensions. As a result of this difference in methodology diameters and volumes cannot be compared 1:1.

We found that older age and hypertension were associated with worse diastolic function at 4 months but not with diastolic remodeling in a 4-month interval after STEMI. This suggests that the effects of age and hypertension might have already affected diastolic function parameters in the first measurement. In epidemiological studies in the general population, age and hypertension have previously been observed to be independent risk factors for deterioration of diastolic function [9, 10].

A major strength of our study is that diastolic function was a predefined secondary endpoint of GIPS-III for the trial and echocardiography was focused on obtaining diastolic parameters which were evaluated by a blinded observer in a core laboratory. Furthermore, we previously suggested that the experimental metformin treatment had no beneficial effect on diastolic function [14]. However, there are some limitations to be acknowledged. Assessment of diastolic function is complex and ideally favors a broader examination than E/eˈ alone. The variability in infarct size was limited by the selection of the population (first STEMI) and the short time between onset of symptoms and PCI. In addition, patients with known diabetes were excluded. Patient enrollment for this study occurred ten years prior to this publication. Treatment of STEMI as well as the assessment of diastolic function have undergone some minor changes since then, potentially limiting the comparability of our results with current practice. Our study would have benefitted from additional analyses into the association of changes in diastolic function with long term clinical outcomes of interest such as all cause death, and heart failure hospitalization. However, we were limited in this regard by an insufficient incidence rate of these outcomes in our study cohort to perform a meaningful analysis.

## Conclusion

Our data supports the hypothesis that female gender and multivessel coronary artery disease are important predictors of adverse diastolic remodeling after STEMI. Furthermore, our results implicate microvascular damage of the coronary arteries as a possible contributing factor to adverse diastolic remodeling. In addition, our data suggests that older age and hypertension prior to STEMI contribute to worse pre-existing diastolic function. These findings may carry implications for our understanding of the development of heart failure with preserved ejection fraction (HFpEF) in patients after STEMI,

## Supplementary Information

**Additional files 1: Table S1.** Discharge medication of all patients, and stratified by E/e′ below and above median at 4 months after STEMI. E/e′: ratio of transmitral flow velocity (E) to early mitral annulus velocity (e′).

## Data Availability

The dataset supporting the conclusions of this study is available through correspondence with the corresponding author.

## Abbreviations

A: Active atrial filling of the left ventricle
ALT: Alanine aminotransferase
AST: Aspartate aminotransferase
CK-MB: Myocardial band creatine kinase
CK-Total: Total creatine kinase
DT: E wave deceleration time
E: Passive early filling of the left ventricle
E/e′: Ratio of transmitral early flow to early mitral annulus velocity
eGFR: Estimated glomerular filtration rate
HbA_1c_: Glycated hemoglobin
HDL: High density lipoprotein
IVRT: Isovolumetric relaxation time
IVS: Interventricular septal wall thickness
IQR: Interquartile range
LAVI: Left atrial volume indexed for body mass
Lateral e′: Early diastolic tissue velocity from lateral wall
LDL: Low density lipoprotein
LV mass: Left ventricular mass
LVEDD: Left ventricular end-diastolic diameter
LVEDV: Left ventricular end-diastolic volume
LVEF: Left ventricular ejection fraction
LVESD: Left ventricular end-systolic diameter
LVESV: Left ventricular end-systolic volume
LVMI: Left ventricular mass indexed for body mass
LVPW: Left ventricular posterior wall thickness
MBG: Myocardial blush grade
MI: Myocardial infarction
NT-proBNP: N-terminal prohormone of brain natriuretic peptide
PCI: Percutaneous coronary intervention
SD: Standard deviation
Septal e′: Early diastolic tissue velocity from septal wall
STEMI: ST-elevation myocardial infarction
TIMI: Thrombolysis in myocardial infarction
UMCG: University Medical Center Groningen
