# Supplementary material for: Predictors of adverse diastolic remodeling in non-diabetic patients presenting with ST-elevation myocardial infarction
Source: BMC Cardiovasc Disord. 2023 Jan 23;23:44. doi: 10.1186/s12872-023-03064-7 (PMC9872414; doi:10.1186/s12872-023-03064-7)
Supplement: Supplementary file 1 — Additional files 1: Table S1. Discharge medication of all patients, and stratified by E/e′ below and above median at 4 months after STEMI. E/e′: ratio of transmitral flow velocity (E) to early mitral annulus velocity (e′). [file 12872_2023_3064_MOESM1_ESM.docx]

**Supplemental table 1.** Discharge medication of all patients, and stratified by E/e' below and above median at 4 months after STEMI. E/e': ratio of transmitral flow velocity (E) to early mitral annulus velocity (e')

|  |  | **No.(%)** |  |  |
| --- | --- | --- | --- | --- |
| **Medication** | **Total (n=267)** | **E/e' ≤ median (n=134)** | **E/e' > median (n=133)** | ***P*-value** |
| Thienopyridine | 267 (100.0%) | 134 (100.0%) | 133 (100.0%) |  |
| Clopidogrel | 189 (70.8%) | 90 (67.2%) | 99 (74.4%) | 0.19 |
| Prasugrel | 3 (1.1%) | 1 (0.7%) | 2 (1.5%) | 0.56 |
| Ticagrelor | 75 (28.1%) | 43 (32.1%) | 32 (24.1%) | 0.14 |
| Aspirin | 260 (97.4%) | 130 (97.0%) | 130 (97.7%) | 0.71 |
| Coumarine | 13 (4.9%) | 6 (4.5%) | 7 (5.3%) | 0.77 |
| Beta-blocker | 255 (95.5%) | 126 (94.0%) | 129 (97.0%) | 0.24 |
| ACE-inhibitor or ARB | 218 (81.6%) | 106 (79.1%) | 112 (84.2%) | 0.28 |
| Calcium-channel blocker | 8 (3.0%) | 4 (3.0%) | 4 (3.0%) | 0.99 |
| Aldosteron receptor antagonist | 25 (9.4%) | 11 (8.2%) | 14 (10.5%) | 0.52 |
| Diuretic | 5 (1.9%) | 2 (1.5%) | 3 (2.3%) | 0.65 |
| Statin | 266 (99.6%) | 134 (100.0%) | 132 (99.2%) | 0.31 |
